# Critical Review of Volatile Organic Compound Analysis in Breath and In Vitro Cell Culture for Detection of Lung Cancer

**DOI:** 10.3390/metabo9030052

**Published:** 2019-03-18

**Authors:** Zhunan Jia, Abhijeet Patra, Viknish Krishnan Kutty, Thirumalai Venkatesan

**Affiliations:** 1 NUSNNI-Nanocore, National University of Singapore, Singapore 117411, Singapore; nnijz@nus.edu.sg (Z.J.); abhijeet@nus.edu.sg (A.P.); viknish@nus.edu.sg (V.K.K.); 2 NUS Graduate School for Integrative Sciences and Engineering, National University of Singapore, Singapore 117456, Singapore; 3 Department of Electrical Engineering, National University of Singapore, Singapore 117583, Singapore; 4 Department of Materials Science and Engineering, National University of Singapore, Singapore 117574, Singapore; 5 Department of Physics, National University of Singapore, Singapore 117581, Singapore

**Keywords:** volatile organic compound, lung cancer, breath analysis, in vitro study, biomarker

## Abstract

Breath analysis is a promising technique for lung cancer screening. Despite the rapid development of breathomics in the last four decades, no consistent, robust, and validated volatile organic compound (VOC) signature for lung cancer has been identified. This review summarizes the identified VOC biomarkers from both exhaled breath analysis and in vitro cultured lung cell lines. Both clinical and in vitro studies have produced inconsistent, and even contradictory, results. Methodological issues that lead to these inconsistencies are reviewed and discussed in detail. Recommendations on addressing specific issues for more accurate biomarker studies have also been made.

## 1. Introduction

Cancer is the second leading cause of death by disease worldwide, exceeded only by heart disease [1]. Among all types of cancer, lung cancer accounts for 1.6 million deaths each year, exceeding those of the next three most common cancers combined (prostate, breast, and colon cancer) [2]. Lung cancer is typically silent in its early stages; symptoms such as coughing, chest pain, weight loss, etc. are often ignored by patients as typical signs of the onset of old age. Histologically, lung cancer is divided into non-small cell lung cancer (NSCLC) and small cell lung cancer (SCLC), with the former accounting for about 85% of cases and the latter, the remaining 15%. NSCLC can further be classified into adenocarcinoma, squamous cell carcinoma, and large cell carcinoma [3]. Treatment options and prognosis are critically dependent on the stage and histology of the disease. Using current diagnostic techniques, such as computer tomography (CT), sputum cytology, and biopsy, 85% of lung cancer cases are diagnosed at a stage when treatment is ineffective at curing the disease [4]. Overall, the 5-year survival is about 10–15% due to late diagnosis. However, if the disease is diagnosed at stage 1, the 5-year survival increases dramatically to 80% [5]. With lung cancer incidences rising around the world, the need for an early detection tool is both critical and urgent. Breath volatile organic compound (VOC) analysis is one such promising technique.

In the last few decades, extensive effort has been focused on searching for VOC biomarkers for lung cancer, either from the headspace of lung cancer cells or from the exhaled breath of patients. However, both clinical and in vitro studies have failed to produce a consistent and validated list. This review aims to summarize the volatile markers produced by these studies in the last 30 years and discuss the methodological issues that have led to the inconsistencies between different studies.

The most commonly used techniques for VOC analysis include mass spectrometry and sensor technologies. Mass spectrometry-based studies usually produce a list of molecules that could be used as biomarkers, while studies using sensor arrays only produce a pattern without individual compound identification. Though detecting lung cancer using various sensor technologies has produced meaningful and promising results [6,7,8,9,10,11,12,13,14], it is beyond the scope of the current review. Comprehensive reviews on lung cancer VOC studies using sensors could be found elsewhere [15,16].

In this review, we focus on mass spectrometry-based clinical, as well as in vitro, studies that provided individual compound identification. We compare the results from these studies and discuss in detail the methodological issues that have led to the inconsistencies across different studies. A short and concise review on breath analysis of lung cancer can be found in [17]. Saalberg et al. and Hua et al. did systematic reviews on breath analysis as a screening technique for lung cancer [18,19]. Zhou et al. discussed the recent developments in the analytical techniques of breath analysis for lung cancer detection [20]. None of these reviews discussed in vitro studies or evaluated the methodological issues of these studies.

## 2. VOC Biomarkers of Lung Cancer in Exhaled Breath

The pioneering study on VOC in exhaled breath from lung cancer patients was done by Gordon et al. in 1985 using gas chromatography mass spectrometry (GC-MS) [21]. Since then, interest in the clinical diagnostic potential of breath analysis in lung cancer detection has risen, evidenced by a rapidly increasing number of publications in the last 30 years. In Table 1, we summarize 25 clinical studies on the breath analysis of lung cancer patients who have identified biomarkers. A majority of these studies adopted a case control approach. Lung cancer patients were recruited as a case group, subjects not clinically diagnosed with lung cancer were recruited as a control group, and the breath VOC profile was compared between them. An identified VOC is considered as a biomarker if its concentration is statistically different between these two groups. Almost all studies used GC-MS as the analytical platform, with the exception of two studies that used proton transfer reaction mass spectrometry (PTR-MS) [22] and ion mobility mass spectrometry (IMS) [23]. Bajtarevic et al. reported results from both PTR-MS and GC-MS [24].

**Figure 1:**
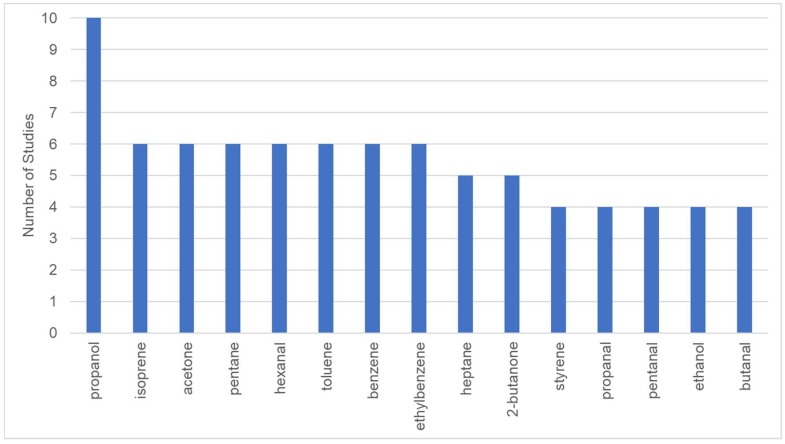
Volatile organic compounds (VOCs) identified as lung cancer biomarker in four or more studies.

The lung cancer biomarkers identified by these studies are largely inconsistent. To better illustrate the biomarker results of Table 1, we filtered the biomarkers that have been identified by at least four studies and ranked them based on the occurrence (Figure 1). The most frequently emerging biomarkers of lung cancer include propanol, isoprene, acetone, pentane, hexanal, toluene, benzene, and ethylbenzene. Michael Philips, one of the pioneers in the breath research field, conducted three independent biomarker discovery studies for lung cancer using GC-MS [25,26,27]. In view of the different lists produced from these studies, he commented that although the exact identities of markers derived from these three studies are not the same, the major biomarkers were mainly alkane derivatives, which are consistent in all three of his studies. The relative abundance of most of these VOCs was found to have decreased in the participants with lung cancer, as compared to the healthy control; this difference could be attributed to the increased catabolism of lipid peroxidation products due to the activated CYP450 genotypes in lung cancer [26]. However, there are many other studies in which alkanes were not found to be associated with lung cancer [28,29,30]. None of these studies evaluated the origin of the detected VOCs. In fact, the mechanism of most VOCs in exhaled breath remains unknown. Hakim et al. reviewed the possible biochemical pathways of lung cancer related VOCs [31].

Generally, it is accepted that until now there has been no consistent and validated list of VOC biomarkers for lung cancer in the literature [31,32,33]. Reasons for these inconsistencies are manifold. There is a large variation in different studies in terms of breath sampling procedures, study designs (selection of control group, selection of patients, etc.), and data analysis protocols. Insightful accounts on the advantages and drawbacks of various data analysis techniques can be found in [34,35].

**Table 1:** Identified volatile biomarkers of lung cancer through breath (Chronological order).

Year	First Author	Sample Size		Biomarker
		Lung Cancer	Control	
**1985**	Gordon [21]	12	17	acetone, 2-butanone, n-propanol
**1988**	O’Neill [36]	8	0	hexane, 2-methylpentane, trimethyl heptane, isoprene, benzene, toluene, ethylbenzene, cumene, trimethyl benzene, alkylbenzene, styrene, naphthalene, 1-methylnaphthalene, propanal, acetone, 2-butanone, phenol, benzaldehyde, acetophenone, nonanal, ethyl propanoate, methyl isobutanoate, dichloromethane, dichlorobenzene, trichloroethane, trichlorofluoromethane, tetrachloroethylene
**1999**	Philips [25]	60	48	styrene, 2,2,4,6,6-pentamethylheptane, 2-methylheptane, decane, n-propylbenzene undecane, methyl cyclopentane, 1-methyl-2-pentylcyclopropane, trichlorofluoromethane, benzene, 1,2,4-trimethylbenzene, isoprene, 3-methyloctane, 1-hexene, 3-methylnonane, 1-heptene, 1,4-dimethylbenzene, 2,4-dimethylheptane, hexanal, cyclohexane, 1-methylethenylbenzene, heptanal
**2003**	Philips [26]	178	102	butane, 3-methyltridecane, 7-methyltridecane, 4-methylctane,3-methylhexane, heptane, 2-methylhexane, pentane, 5-methyldecane
**2005**	Poli [37]	36	85	2-methylpentane, pentane, ethylbenzene, xylenes, trimethylbenzene, toluene, benzene, heptane, decane, styrene, octane, pentamethyl heptane
**2007**	Philips [27]	193	211	1,5,9-trimethyl-1,5,9-cyclododecatriene, 2,2,4-trimethyl-1,3-pentanediol tributyrate, ethyl 4-ethoxybenzoate, 2-methyl- propanoic acid, (1,1-dimethylethyl)-2-methyl-1,3-propanediyl ester, 10,11-dihydro-5H-dibenz-(b,f)-azepine, 2,5-2,6-bis(1,1-dimethylethyl)-cyclohexadiene-1,4-dione, 1,1-oxybi-benzene, 2,5-dimethyl-furan, 2,2-diethyl-1,1-biphenyl, 2,4-dimethyl-3-pentanone, trans-caryophyllene, 2,3-dihydro-1,1,3-trimethyl-3-phenyl-1H-indene, 1-propanol, 4-methyl-decane, 1,2-benzenedicarboxylic acid, diethyl ester, 2,5-dimethyl-2,4-hexadiene
**2007**	Wehinger [38]	17	170	formaldehyde, isopropanol
**2009**	Bajtarevic [24]	220	441	isoprene, acetone, methanol, 2-butanone, benzaldehyde, 2,3-butanedione, 1-propanol
**2010**	Fuchs [28]	12	12	pentanal, hexanal, octanal, nonanal
**2010**	Peng [39]	30	22	p-cymene, toluene, dodecane, 3,3-dimethylpentane, 2,3,4-trimethylhexane, (1-phenyl-1-butenyl)benzene 1,3-dimethylbenzene, 1-iodononane, [(1,1-dimethylethyl) thiol]acetic acid, 4-(4-propylcyclohexyl)-4′-cyano[1,1′-biphenyl]4-yl ester benzoic acid, 2-amino-5-isopropyl-8-methyl-1-azulenecarbonitrile, 5-(2-methylpropyl)nonane, 2,3,4-trimethyldecane, 6-ethyl-3-octanyl 2-(trifluoromethyl)benzoate, p-xylene, and 2,2-dimethyldecane
**2010**	Song [40]	43	41	1-butanol, 3-hydroxy-2-butanone
**2010**	Kischkel [41]	31	31	isoprene, acetone, 2-butanone, cyclohexanone, dimethyl sulfide, acetonitrile, ethanol, isopropanol, acetaldehyde, propanal, butanal, pentanal, hexanal, heptanal, octanal, 2-propenal, 2-butenal, propane, butane, pentane, hexane, heptane, 2-methylbutane, 2-methylpropanal, 2,2-dimethylbutane, 2,3-dimethylbutane, 2-methylpentane, 3-methylpentane, 2,2-dimethylpentane, 2,4-dimethylpentane, 3,3-dimethylpentane, 2-methylhexane, cyclohexane, benzene, toluene, chlorobenzene, 1,2-dimethylbenzene, 1,2-dichlorobenzene, carbon disulfide, dimethyl formamide, 2,5-dimethylfuran, 1-propanol
**2011**	Rudnicka [42]	23	30	propane, carbon disulfide, 2-propenal, ethylbenzene, isopropyl alcohol
**2011**	Ulanowska [43]	134	143	ethanol, acetone, butane, dimethyl sulfide, isoprene, propanal, 1-propanol, 2-pentanone, furan, o-xylene, ethylbenzene, pentanal, hexanal, nonane
**2012**	Peled [44]	53	19	1-octene
**2012**	Wang [45]	88	85	2,4,6-trimethyloctane, 2-methyldodecane, 2-tridecanone, 2-pentadecanone, 8-methylheptadecane, 2-heptadecanone, nonadecane, eicosane
**2012**	Buszewski [46]	29	44	butanal, ethyl acetate, 2-pentanone, ethylbenzene, 1-propanol, 2-propanol
**2014**	Handa [23]	50	39	3-methyldodecane, 1-butanol, 2-methylbutylacetate/2-hexanol/nonanal cyclohexanone, isopropylamine, ethylbenzene, hexanal, cyclohexanone, heptanal, 3-methyl-1-butanol
**2014**	Wang [29]	18	0	caprolactam, propanoic acid
**2014**	Zou [47]	79	38	2-methyl-5-propylnonane, butylated hydroxytoluene, 2,6,11-trimethyl-dodecane, hexadecanal, 8-hexylpentadecane
**2015**	Capuano [48]	20	10	ethanol, 2-butanone, thiophene, 4-heptanone, butanoic acid, , acetic acid, cyclohexanone, 2,2,-dimethyl-hexanal, 1,1-diethoxy-3-methylbutane; 1-(1-ethoxyethoxy)-pentane, 2,2,6-trimethyloctane, 2-ehtyl-1-hexanol, undecane, thymol, 2-methyl-1-decanol, 3,7-dimethyl- decane,
**2015**	Corradi [49]	71	67	pentane, 2-methylpentane, hexane, benzene, ethylbenzene, trimethylbenzene, heptane, pentamethyl heptane, toluene, total xylenes, styrene, propanal, butanal, pentanal, hexanal, heptanal, octanal, nonanal, trans-2-hexenal, trans-2-heptenal, trans-2-nonenal
**2016**	Monila [30]	68	60	p-cresol, eicosenamide, 1-hexadecylindane and cumyl alcohol
**2016**	Schallschmidt [50]	37	23	propanal, butanal, decanal, butanal, 2-butanone, ethylbenzene
**2017**	Sakumura [51]	107	29	hydrogen cyanide, methanol, acetonitrile, isoprene, 1-propanol

### 2.1. Methodological Issues of Clinical Studies

In Table 2, we have summarized and listed the various methodological issues in breath sampling and study design. In this section, we will discuss in detail the effect of various methodological issues, what researchers have done in the included 25 studies to account for these issues, and, consequently, what the current best practices are, based on these studies.

### 2.2. Environmental VOCs

More than 1000 VOCs have been detected in human breath and the majority of these VOCs have exogenous origins [52]. The effect of environmental VOCs on breath analysis was first recognized by Philips, and he has proposed that this problem can be solved by determination of the “alveolar gradient” of a VOC [42,43]. The alveolar gradient is defined as the concentration of the VOC in breath minus the concentration in the room air. A positive alveolar gradient means more of the VOC was exhaled than inhaled and vice versa. Philips measured the alveolar gradients of various VOCs and concluded that VOCs with negative alveolar gradients are metabolized by the body and those with positive alveolar gradients are manufactured in the body [53,54]. However, later studies proved this to be an incorrect assertion. VOCs with positive alveolar gradients may result from VOCs absorbed from food [55], drugs [56,57], or even bacteria in the GI tract, airways, or mouth cavity [58]. On the other hand, VOCs with a negative alveolar gradient may, in fact, have metabolic origins. The journey of environmental VOCs in the human body is a complex process of mixing, diffusion, distribution in blood and fat tissues, and metabolism, as shown in the report by Philips et al. [59]. The rate and degree to which environmental VOCs are removed from the body depend on the concentration of the VOC, the duration of exposure [60], the solubility in blood and lipid tissues [61], and individual physiology.

Early theoretical modelling experiments, aimed at evaluating the health effects of industrial VOC exposure, have shown that the partition coefficient of a VOC in lungs, blood, and tissue is specific to its physical and chemical properties, and varies immensely [62,63]. Schubert et al. measured inspired, expired, and blood concentrations for four VOCs (pentane, acetone, isoprene, and isoflurane) and found that only when the inspired concentration was less than 5% of the expired concentration did the disappearance rate of VOC from the blood correlate significantly with the rate of exhalation [64]. Another study by Spanel et al. found that all seven studied VOCs (pentane, isoprene, acetone, ammonia, methanol, formaldehyde, and deuterated water) are partially retained by the body, and there are close linear relationships between the exhaled and inhaled concentrations [65]. They also introduced a useful parameter called a retention coefficient, which is the ratio of the increase of the exhaled concentration to the increase of the inhaled concentration. The retention coefficients measured for these seven VOCs vary numerically from 0.06 for formaldehyde to 0.76 for pentane [65].

With these initial efforts, it is evident, unfortunately, that there is no general rule that can be applied to all VOCs when accounting for the effects of environmental VOCs. Apart from using the concept of alveolar gradient, researchers have addressed this issue either by using an inspiratory filter [12] or by letting the patients stay in a ventilated room for a predetermined amount of time before collection [45].

For discovery-type studies, it seems that an inspiratory filter is the best solution, for now. However, the time it takes to clear out environmental VOCs from the body is compound specific. Much more effort is, therefore, needed to understand the origins and dynamics of various VOCs observed in human breath.

### 2.3. Phase of Breath Sample Collected

Each exhalation can be divided into three phases based on the CO_2_ pressure in the breath. Phase 1 and phase 2 are air from the dead space in oral cavity and upper airways. Phase 3 is alveolar air from deep inside the lungs [66]. End-tidal breath refers to the portion of alveolar air nearer to the end of one exhalation. For the purpose of disease diagnostics, the alveolar phase is desired because VOCs in this portion are from the blood-gas exchange in the alveoli and thus more closely reflect metabolic conditions. Concentrations of certain VOCs differ in whole breath versus end-tidal breath. For example, VOCs such as carbonic acid, dimethyl ester, and methyl format were found to be significantly higher in end-tidal breath, while methylene chloride and 3-ethyl pentane were lower in end-tidal breath than in whole breath [67].

Most studies on lung cancer breath analysis either collected whole breath [26,38] or collected alveolar or end-tidal breath based on a crude estimation. The end-tidal breath was collected either by discarding the front portion of the breath [37,45] or by filling the dead space air into separate bags [39]. Kischkel et al. collected alveolar breath based on a fast responding CO_2_ sensor [41].

To selectively collect alveolar phase accurately, monitoring CO_2_ pressure is a good idea. CO_2_ level is a reliable indicator of the phase, and CO_2_ sensors are readily available. Birken et al. integrated a capnography setup into the breath collection procedure to visually monitor the phase of breath and manually draw alveolar air using a syringe [68]. Later, the same group developed an automatic CO_2_ controlled sampling device and demonstrated the performance of the automatic sampler to be comparable to manual sampling [69]. In 2016, Owlstone Medical developed a breath collection device named RECIVA™ [70]. This is the first and only commercially available breath collection device that allows an accurate selection of phase. Such controlled and standardized methods in selecting phase of breath could significantly improve the consistency of breath biomarker research.

### 2.4. Expiratory Flow Rate, Breath-Holding, and Hyperventilation

Studies have shown that expiratory flow rate and hyperventilation affect the levels of various VOCs. Contradictory results were reported on the effect of flow rates on common breath VOCs. Doran et al. found that at a higher flow rate, lower levels of acetone and phenols were observed [67]. However, in another study, acetone level was not affected by the expiratory flow rate [71], and in yet another study, higher levels of acetone at a higher flow rate were reported [72]. Reports on trends of isoprene at different expiratory flow rates are also contradictory [73,74].

When asked to provide a breath sample, some people tend to hold their breath before exhalation. Therefore, the effect of breath-holding on breath VOC was investigated, and results from different studies are in agreement. Pentane and isoprene levels increased significantly after 20 s of breath-bolding [73]. A similar trend was observed in another study for isoprene after a 40 s breath holding [74]. Other VOCs found to be increased after breath holding in this study include 2-propanol and acetaldehyde [74]. In a third study, acetone, methanol, isoprene, and dimethyl sulphide increased significantly after 30 s of breath holding [72]. The unanimous increase of various VOCs with breath holding may be attributed to the prolonged time for them to diffuse from the alveoli to the airway.

Hyperventilation was found to have a negative effect on levels of methanol, dimethyl sulfide, acetaldehyde, and ethanol [72], as well as isoprene [75]. On the other hand, acetone level was not notably affected by hyperventilation [75]. This is attributed to the fact that acetone has much lower solubility in blood than the rest of the VOCs, and, therefore, can be quickly released from blood during hyperventilation.

The diameter of the mouthpiece used during sampling affects airway resistance and, subsequently, affects levels of certain VOCs. It was found that a smaller mouthpiece diameter caused a 19% increase in isoprene levels. Furan hydrogen sulfide also increased significantly [76]. A mouthpiece with a diameter larger than 1 cm was recommended for future studies [76].

In all the studies included in Table 1, subjects were asked to breath “normally”, flow rate was not measured, and no information on the diameter of mouthpiece was given.

The learning outcome from these studies is that sampling parameters, such as exhalation rate, breath-holding, and airway resistance, must be controlled and recorded in a standardized manner across different subjects for consistent and reliable results. Hyperventilation should be avoided.

### 2.5. Temperature and Humidity of Environmental Air

This is a seldom noticed, but important, confounder for longitudinal studies, in which breath collection spans over a long period of time, during which temperature and humidity change drastically (e.g., winter to summer) [44]. This factor is also critical for multi-center clinical studies, when collection is done in different regions of the world with significantly different climates [26]. Thekedar et al. [74] assessed the changes in exhaled VOC concentrations sampled after a 5 min stay under 3 °C, 47% relative humidity and 27 °C, 19% relative humidity using PTR-MS. Acetonitrile, ethanol, methanol, and propanol showed higher concentrations in the samples collected under the warm air compared to those collected in the cold air. On the other hand, VOCs with a proton transfer reaction product of ion *m*/*z* s of 85, 86, 99 and 169 showed higher concentrations in samples collected under cold air. The fact that studies on lung cancer biomarkers were conducted under vastly different temperature and humidity conditions is another significant reason for the inconsistent results.

### 2.6. Contamination from Collection Systems

Unfortunately, most commercially available breath collection apparatuses involve materials that could, themselves, be sources of contamination. For example, the widely accepted Tedlar bags for breath collection are made of polyvinyl fluoride and are known to emit many VOCs from the bag material, which, therefore, poses a threat of contamination [77,78]. Several studies used tedlar bags for lung cancer biomarker discovery [38,39,42,47]. Inert materials such as Teflon, stainless steel, or glass should be used for breath collection systems and other types of materials should be avoided as much as possible.

### 2.7. Age/Gender

Isoprene, alkanes and methylated alkanes were found to be related to age [79,80,81]. With an increase in oxidative stress level with advancing age, levels of these VOCs in breath increase gradually. Spanel et al. also found that breath ammonia increases with increasing age, but acetone and hydrogen cyanide do not vary greatly with age [82]. Gender also influences the breath VOC profile. isoprene and several other VOCs were found to be gender specific [41,80,83].

In case control studies, results could be biased due to unmatched age and gender. In the reviewed studies, lung cancer patients are often significantly older than control subjects [23,24,38,41,43,44,45,47], and usually there are more male subjects in the case group than in the control group [24,26,41,43].

### 2.8. Diet

The effect of diet on breath VOCs is also a complex one. Certain types of food, such as yoghurt [84] and seafood [85] contains a number of VOCs that rapidly and directly appear after ingestion. Food also affects breath VOC by changing metabolism, inflammation, or redox status, or by interacting with gut flora. Some studies required subjects to fast for 12 h, or overnight, before breath collection [25,42,45,47], while other studies had no restrictions; It is not understood how long it takes for the VOCs from diet to be eliminated from breath. Whether overnight fasting helps in eliminating these effects needs to be studied further. On the other hand, dietary style may have a prolonged effect that could not be eliminated by fasting.

### 2.9. Smoking

Smoking was identified as one of the key risk factors for lung cancer, and smoke contain many VOCs. It was found smokers have higher levels of benzene and acetonitrile in their breath. Although the level of benzene in a smoker rapidly decreases to a similar level as a non-smoker within an hour, the level of acetonitrile, since the last smoking, takes about a week to become that of a non-smoker [86]. Alcohol consumption leads to increased levels of acetaldehyde in the breath, and it was found long-term smoking elevates the production of acetaldehyde from alcohol [56]. This finding was confirmed by another study [87]. These results show that smoking can affect other metabolic pathways that are not directly related to VOCs from cigarettes. Smoking cigarettes is also known to increase oxidative stress. As a result, levels of isoprene and pentane were found to be increased after smoking [88]. Other smoking-related VOCs include 2,5-dimethyl furan and 1,3-butadiene. Smoking related VOCs need to be clearly distinguished from endogenous compounds that are related to disease conditions.

All studies reported the smoking history of recruited subjects but adopted vastly different strategies for data analysis. Some studies did not discuss the possible effects of smoking on their results, even though the case and control group had highly uneven smoking histories [40,47]. Coraddi et al. found the value “pack-year” alone had a fair diagnostic power [49]. Combining this value with VOC markers could help in developing a more robust biomarker panel for lung cancer detection. Wang et al. identified smoking related VOCs using ROC and excluded these molecules from the lung cancer biomarker list [45].

Currently, there are two ways to minimize the influence of smoking. One is to design the study carefully, so that case and control groups have matched smoking histories. In two studies where the smoking histories of the case and control groups were closely matched, no effect of tobacco smoke was found on the diagnostic power of the identified biomarker panel [26,27]. The other strategy is to exclude smoking-related VOCs from the biomarkers for lung cancer detection. However, it is not fully known yet what other metabolic pathways may be affected by smoking.

### 2.10. Comorbidity

Many target subjects for disease diagnostic studies often have more than one medical condition. These diseases will also change the VOC profile and confound the biomarker discovery for the targeting disease. Most studies recruited healthy subjects as a control group [29,41,50], while a few studies recruited subjects with similar comorbidity as a control group [44]. The variances in the choice of control groups contribute to the inconsistencies across the different studies.

### 2.11. Disease Staging

One key advantage of breath analysis is its potential for early detection. It is of keen interest to know if stage influences VOC profile. Philips et al. identified 22 breath VOCs that could differentiate control and lung cancer subjects, regardless of stages. For stage 1 patients, the 22 VOCs had 100% sensitivity and 81.3% specificity [25]. Other studies also showed no discrimination between early stage and advanced stage [26,38,40,45,47]. However, Corradi et al. showed that although lung cancer patients have higher levels of ethylbenzene in their breath, the difference is less pronounced between early stage lung cancer and control subjects [49]. Peled et al. analyzed breath samples using a combination of GC-MS and chemical nanoarray, GC-MS analysis did not show any discrimination between early stage and late stage, and also for sub-histological types of lung cancer; however, chemical nanoarray-based techniques could discriminate between early and late stage, and between adenocarcinoma and squamous cell carcinoma, with an accuracy of 88% [44]. Due to the limitations of the chemical sensor array, the identity of the VOCs that contribute to these discriminations is unknown.

### 2.12. Histology

Lung cancer is a complex disease with different histologies. Very few studies compared the VOC profile between different histological types. Song et al. found that patients with adenocarcinoma showed higher concentrations of 1-butanol and 3-hydroxy-2-butanone [40]. In the study by Corradi et al., adenocarcinoma showed higher levels of hexane and ethyl benzene compared to squamous cell carcinoma [49]. Other studies showed that histology has no significant impact on breath VOCs [26,27,47].

Most of the methodological issues discussed above are not limited to lung cancer. Rather, they are shared by breath VOC studies with various objectives. Establishing a standardized practice for these methodological issues is a challenging task and requires a collective effort from all researchers in the field. Jens et al. suggested a framework for standardizing breath analysis at different technical levels [89]. In 2017, the European Respiratory Society published a technical standard on exhaled biomarkers in lung disease [90] and highlighted a few key areas for future research. These are important first steps towards standardized protocols in breath analysis. For highly complex and heterogenous diseases, such as lung cancer, implementing standardized practice is especially critical in developing biomarkers with a clinical value. Though much more needs to be done to establish a standardized methodological procedure, this area of study is well worth pursuing due to the huge potential of breath analysis for non-invasive and early disease detection. 

## 3. In Vitro Studies

In vitro cell culture provides a convenient alternative for studying volatile signatures of lung cancer while bypassing many confounding factors associated with breath sampling. Many studies have identified the VOC biomarker of cultured lung cells, and the results show that different types of lung cell lines can generate different panels of VOCs (Table 3). Studies from the same cell line using different techniques produced inconsistent results. For example, a study of the NSCLC cell line Calu-1 using selected ion flow tube-mass spectrometry (SIFT-MS) [91,92,93] consistently showed higher levels of acetaldehyde, while a study by GC-MS [94] showed that acetaldehyde was decreased in this cell line. Sporning et al. also found decreased level of acetaldehyde in another type of lung cancer cell line [95]. Most studies used only one or two cell lines. Two studies included more than six cell lines [96,97]. These studies brought in vitro studies one step further to investigating whether VOCs from cells in vitro could discriminate between different histologies. Barash et al. showed that VOCs could discriminate between (1) lung cancer and normal lung epithelial cells; (2) NSCLC and SCLC cells; and (3) two subtype of NSCLC: adenocarcinoma and squamous cell carcinoma [97]. Jia et al. demonstrated that although NSCLC and SCLC showed distinct VOC profiles, adenocarcinoma and squamous cell carcinomas could not be differentiated among NSCLCs. On the other hand, large cell carcinomas show different VOC profile with the rest of the NSCLCs [97].

### Limitations of In Vitro Studies

Though analyzing VOCs from cultured cells faces fewer problems compared to analyzing human breath samples, there are many methodological issues in the current literature.

The first issue is that almost all studies have used standard cell culture flasks made of polystyrene. Polymer materials like polystyrene emit VOCs themselves. The background from the vessel should be measured and corrected in cell experiments. Alternatively, if cells can survive, glass vessels should be used instead, as adopted by some studies [91,102]. Schallschmidt et al. measured the background from plastic culture vessels and identified several alkanes and aromatics [103]. These molecules are often also found in cultures with living cells. As a result, the background from plastic cell culture vessels may easily lead to misinterpretations.

The second issue is that different cell growth media other than those recommended by the supplier were used to get a uniform VOC background. A culture medium contains nutrients, such as glucose, amino acids, and vitamins, that are essential to cell growth. Certain cell lines require special formulations for optimum growth. The most commonly used basic medium is called DMEM (Dulbecco’s modified Eagle’s medium). A culture medium has a considerable VOC background and differs from one type to another. In some studies, a cell line with a special medium requirement was cultured in a basic medium, in order to get the same VOC background across different cell lines. Filipiak el al. studied three cell lines: lung cancer cell line A549, primary human bronchial epithelial cells (HBEpC), and human fibroblasts (hFB) [104]. Although the authors cultivated HBEpC in an airway epithelial cell growth medium with special supplements, as recommended by the ATCC (American Type Culture Collection) for initial propagation, for the VOC experiment they cultured all three types of cells in DMEM for 21 h. It is questionable whether the HBEpC cells remained in a healthy condition in DMEM, as no cell viability data or pictures of the cells were shown. It is beyond doubt that different VOC backgrounds from different types of cell growth media should be taken into consideration. Instead of compromising on the growth condition of cells, we believe the method adopted by Barash et al. is more acceptable [96], where each cell line was grown in its recommended medium and the VOC effect of the medium was corrected during data analysis before comparing across different cell lines.

Another limitation is that cells in an in vitro culture live in a drastically different environment than tumor cells in the human body. As a result, none of the identified biomarkers achieved clinical relevance [105,106]. Kalluri et al. showed that hypoxia influences the VOCs that the cancer cells produce and suggested future in vitro studies to culture cells in hypoxic conditions [106]. Lung cancer cell grown in a 3D environment was found to emit higher levels of VOCs than in 2D cultures [93]. These studies indicate that cell culture experiments could be more relevant when the conditions better mimic the real situation.

Despite these limitations, in vitro cell culture provides a convenient way to directly assess the effect of certain stimuli on the VOC profile produced by cancer cells. Lawal et al. used cultured lung cells to study the effect of a bacterial infection on the VOC profile [107]. They co-cultured lung epithelial cells with *Pseudomonas aeruginosa*, a bacterium commonly found in pneumonia, and measured the VOC profile with and without the bacteria. Acetone, ethanol, 3-methyl-1butanol, and three other VOCs were found to be elevated in bacteria infected cells, indicating the bacterial origin of these VOCs. They also simulated the effect of oxidative stress induced by bacterial infection by adding hydrogen peroxide to the cell culture and identified several alkanes as potential markers for oxidative stress. Feinberg et al. blocked glycolysis in cultured lung cancer cells and identified unique signatures in all cells studied [108]. A recent study identified the unique VOC profiles of lung cancer cells with a different p53 mutation status at a single cell level [102]. These studies demonstrated the usefulness of in vitro cell cultures in identifying the possible biochemical origins of VOCs.

## 4. Conclusions

Breath analysis for lung cancer screening is a rapidly developing field. Accelerating the pace of the development of a robust panel of markers that can be translated for clinical use will require progress in three key areas: (1) development of standardized and flexible breath sampling protocols, (2) longitudinal multi-centre clinical trials with careful study design and external validation, and (3) understanding of the biochemical pathways involved in lung cancer development and progression. Measuring VOCs in vitro, after blocking specific pathways or knocking out specific genes, provides direct evidence of the biochemical origins of the VOCs. We believe that these discoveries will ultimately contribute to the development of breath analysis as a technique for the early detection of lung cancer, allowing breath analysis to realize its long-held potential and to become a critical tool in personalized medicine.

## Figures and Tables

**Table 2:** Methodological Issues of Clinical Studies.

Breath Sampling	Study Design
1. Environmental VOCs 2. Phase of breath (alveolar vs. whole breath) 3. Expiratory flow rate and Hyperventilation 4. Temperature and humidity of environment 5. Contaminations from collection system	6. Age/gender 7. Diet 8. Exercise 9. Smoking 10. Medication 11. Comorbidities 12. Disease Stage 13. Histology

**Table 3:** Identified volatile biomarker of lung cancer from in vitro studies.

First Author	Cancer Cell	Normal Cell	Analytical Technique	VOC-Increased Concentration	VOC-Decreased Concentration
**David [91]**	SK-MES and CALU-1		SIFT-MS	acetaldehyde	
**Chen [98]**	primary tissues		SPME-GC-MS	styrene, decane, isoprene and benzene	
**Filipiak [94]**	CALU-1		GC-MS	2,3,3-trimethylpentane, 2,3,5-trimethylhexane, 2,4-dimethylheptane, 4-methyloctane	acetaldehyde, 3-methylbutanal, n-butyl acetate, acetonitrile, acrolein, methacrolein, 2-methylpropanal, 2-butanone, 2-methoxy-2-methylpropane, 2-ethoxy-2-methylpropane, hexanal
**Sule-Suso [92]**	CALU-1	NL20 and 35FL121 Tel+	SIFT-MS	acetaldehyde	
**Sponring [95]**	NCI-H2087		GC-MS	2-ethyl-1-hexanol and 2-methylpenthane	acetaldehyde, 2-methylpropanal, 3-methylbutanal, 2-methylbutanal, hexanal, n-butyl acetate
**Brunner [99]**	A549		PTR-MS	2-pentanone, 2-methyl-1-pentene, 2,4-dimethyl-1-heptene, acetone, ethanol, isobutene, n-octane, tert-butyl methyl ether, tert-butyl ethyl ether	n-butyl acetate, 3-methylbutanal, 2-methylpropanal, methacrolein, 2-methyl-2-butenal, 2-ethylacrolein, pyrrole
**Hanai [100]**	A549		SPME-GC-MS	dimethyl succinate, 2-pentanone, phenol, 2-methylpyrazine, 2-hexanone and acetophenone	benzophenone, maltol, dimethyl disulfide, methanethiol, 1-butanol, acetonitrile, cyclohexanone, tributyl phosphate, 2-methyl-1-propanal, benzyl alcohol, styrene
**Rutter [93]**	CALU-1	NL20	SIFT-MS	acetaldehyde	
**Barash [96]**	H1650, H820, A549, H1975, H4006, H1435, CALU-3, H2009, HCC95, HCC15, H226, NE18, H774, H69, H187, H526	Minna 3KT	GC-MS		decanal
**Wang [45]**	A549, NCI-H446, SK-MES-1	BEAS-2B	GC-MS	2-pentadecanone, nonadecane and eicosane	
**Jia [97]**	A549, HCC827, H226, H520, H460, H526	SAEC	SPME-GC-MS	benzaldehyde, 2-ehtyl-1-hexanol, 2,4-decadien-1-ol	
**Thriumani [101]**	A549, Calu-3	WI38VA13	SPME-GC-MS	decane, ethylbenzene, n-propyl benzene, 1-ethyl-2-methylbenzene, styrene, dodecane, cyclohexanol, decanal, nonanal, 1,3-Di-tert-butylbenzene, tetradecane, 2-ethyl-1-dodecanol, 2-ethylhexanol, benzaldehyde, acetophenone, 2-Ethyl-m-xylene, 1-methyl-2-pyrrolidinone, heneicosane	ethanedioic acid
